
JOURNAL INFORMATION
==============================
NLM Title Abbreviation: ScientificWorldJournal
Journal ID: ScientificWorldJournal
NLM Journal ID: 101131163
Journal ID: TSWJ

The Scientific World Journal

ISSN: 2356-6140
EISSN: 1537-744X
Publisher: Wiley

ARTICLE INFORMATION
==============================
PMCID: PMC6083848
PMID: 30147508
DOI: 10.1100/tsw.2001.182
Article ID: 278158
Article version: 1
Subjects: Short Report

Peripheral Nerve Degeneration in the HIV-1 Transgenic Rat

Denaro F.J. *
Hayes N.
Jones O.
McCready M.
Davis H.
Reid B.
Gallo R.
Bryant J.
Institute of Human Virology, Baltimore, MD, USA
*F.J. Denaro: denaro@umbi.umd.edu
Publication date: 2001
Electronic publication date: 2001 Aug 9
Volume: 1
First page: 50
Last page: 50
Copyright: Copyright © 2001 F.J. Denaro et al.
Copyright year: 2001
License: This is an open access article distributed under the Creative Commons Attribution License, which permits unrestricted use, distribution, and reproduction in any medium, provided the original work is properly cited.
License URL: https://creativecommons.org/licenses/by/3.0/

Funding: National Institutes of Health NS31857 National Institutes of Health MH29494

==============================
Miami *Nature Biotechnology* Short Reports
*TheScientificWorld* (2001) 1 (S3), 50SR
ISSN 1532-2246; DOI 10.1100/tsw.2001.182

### PERIPHERAL NERVE DEGENERATION IN THE HIV-1 TRANSGENIC RAT

F.J. Denaro*, N. Hayes, O. Jones, M. McCready, H. Davis, B. Reid, R. Gallo, and J. Bryant

Institute of Human Virology, Baltimore, MD
* Denaro@umbi.umd.edu

**INTRODUCTION.** Neurological abnormalities of the peripheral (PNS) and central nervous system continue to cause debilitating symptoms in AIDS patients despite advances in antiviral therapy (1). How HIV-1 mediates PNS damage is unknown. Our understanding is limited by the lack of model systems in which HIV/PNS pathology can be studied. Studies on the recently developed HIV-1 transgenic rat (HIV-1TgR) reveal that a subset of these animals displays severe neurological signs. These symptoms include complete or partial paralysis, difficulties in walking and sensitivity to touch. Progressive skeletal muscle mass loss with increasing severity of motor symptoms occurs over time. These clinical observations suggest that morphological changes to the nerve might be found. Peripheral nerve was examined in a group of HIV-1 TGRs which displayed neurologic symptoms. By characterizing the abnormalities in this model, it may be possible to develop theories on pathogenesis relevant to treatment.

**METHODS.** The colony of HIV-1TgRs was monitored over time and HIV-1 TGRs with neurologic abnormalities were selected for further study. The symptoms displayed by these animals included striated muscular atrophy, difficulties in walking, evidence of circling behavior and muscular weakness. These animals together with littermate controls were examined for peripheral nerve changes by light and electron microscopy. The stains used were H&E, Bielschowsky, and Luxol fast blue. Nerve teasing was used to examine individual fibers. Peripheral nerve was taken from the hind forelimbs. Both motor and sensory nerves were sampled.

**RESULTS.** H&E stains reveal multiple areas of inflammation along the length of the nerve. This inflammation may vary from mild to severe. Stains for myelin display small multiple foci of demyelination (Fig. 1). This observation was further confirmed by nerve teasing. Isolated nerve fibers displayed segmental myelin loss (Fig. 2). In addition teased single nerves revealed swellings along their lengths. Bielschowsky stains suggest axonal loss (Fig. 3). Electron microscopic observations of the PNS reveal that both the myelin and axons are undergoing degeneration.

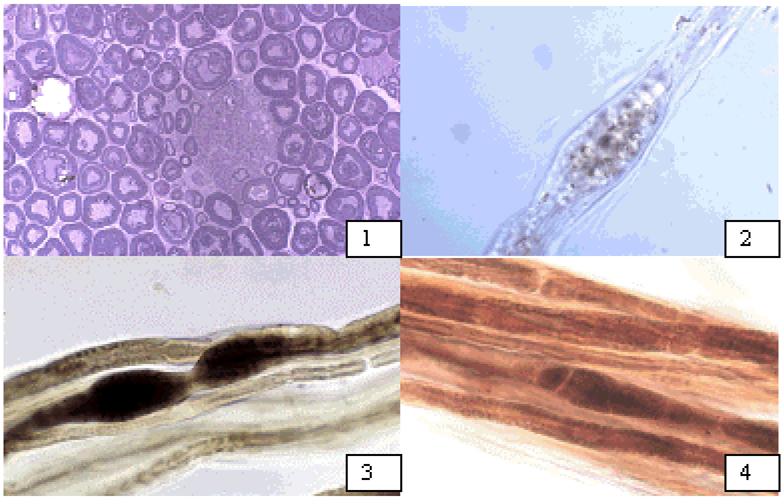

**DISCUSSION.** The HIV-1 TgR displays interesting systemic histopathology that is very similar to AIDS. The observed clinical neurological abnormalities also replicated the neurological symptoms. Consistent with these clinical findings is evidence of axonal degeneration and myelin loss. While the primate model for HIV infection is the most comprehensive model existing today for the study of AIDS, a small, non-infectious model would greatly facilitate research. Moreover a small animal model of HIV/PNS pathology may also offer unique strengths. For example, clinical and behavioral tests can be easily studied in this non- infectious model. As the clinical finding and neuropathology can be quantified, the potential exists for the testing of neuroprotectants or antivirals.

**ACKNOWLEDGEMENTS.** Supported by IHV and NIH NS31857 and MH29494.

**REFERENCE.**

1. Enzensberger, W. (1999) Eur. J. Med. Res. 11, 456-462
